# Enhanced tumor accumulation and therapeutic efficacy of liposomal drugs through over-threshold dosing

**DOI:** 10.1186/s12951-022-01349-1

**Published:** 2022-03-15

**Authors:** Hui Ao, Zhuo Wang, Likang Lu, Hongwei Ma, Haowen Li, Jingxin Fu, Manzhen Li, Meihua Han, Yifei Guo, Xiangtao Wang

**Affiliations:** 1 Institute of Medicinal Plant Development, Chinese Academy of Medical Sciences and Peking Union Medical College, No. 151, Malianwa North Road, Haidian District, Beijing, 100193 People’s Republic of China; 2 College of Pharmacy, Heilongjiang University of Chinese Medicine, No. 24, Heping Road, Xiangfang District, Harbin, 150040 People’s Republic of China

**Keywords:** Over-threshold dosing, Tumor delivery, Annonaceous acetogenins, Liposomes, Antitumor

## Abstract

**Background:**

Most intravenously administered drug-loaded nanoparticles are taken up by liver Kupffer cells, and only a small portion can accumulate at the tumor, resulting in an unsatisfactory therapeutic efficacy and side effects for chemotherapeutic agents. Tumor-targeted drug delivery proves to be the best way to solve this problem; however, the complex synthesis, or surface modification process, together with the astonishing high cost make its clinical translation nearly impossible.

**Methods:**

Referring to Ouyang’s work and over-threshold dosing theory in general, blank PEGylated liposomes (PEG-Lipo) were prepared and used as tumor delivery enhancers to determine whether they could significantly enhance the tumor accumulation and in vivo antitumor efficacy of co-injected liposomal ACGs (PEG-ACGs-Lipo), a naturally resourced chemotherapeutic. Here, the phospholipid dose was used as an indicator of the number of liposomes particles with similar particle sizes, and the liposomes was labelled with DiR, a near-red fluorescent probe, to trace their in vivo biodistribution. Two mouse models, 4T1-bearing and U87-bearing, were employed for in vivo examination.

**Results:**

PEG-Lipo and PEG-ACGs-Lipo had similar diameters. At a low-threshold dose (12 mg/kg equivalent phospholipids), PEG-Lipo was mainly distributed in the liver rather than in the tumor, with the relative tumor targeting index (RTTI) being ~ 0.38 at 72 h after administration. When over-threshold was administered (50 mg/kg or 80 mg/kg of equivalent phospholipids), a much higher and quicker drug accumulation in tumors and a much lower drug accumulation in the liver were observed, with the RTTI increasing to ~ 0.9. The in vivo antitumor study in 4T1 tumor-bearing mice showed that, compared to PEG-ACGs-Lipo alone (2.25 mg/kg phospholipids), the co-injection of a large dose of blank PEG-Lipo (50 mg/kg of phospholipids) significantly reduced the tumor volume of the mice by 22.6% (*P* < 0.05) and enhanced the RTTI from 0.41 to 1.34. The intravenous injection of a low drug loading content (LDLC) of liposomal ACGs (the same dose of ACGs at 50 mg/kg of equivalent phospholipids) achieved a similar tumor inhibition rate (TIR) to that of co-injection. In the U87 MG tumor-bearing mouse model, co-injection of the enhancer also significantly promoted the TIR (83.32% vs. 66.80%, P < 0.05) and survival time of PEG-ACGs-Lipo.

**Conclusion:**

An over-threshold dosing strategy proved to be a simple and feasible way to enhance the tumor delivery and antitumor efficacy of nanomedicines and was benefited to benefit their clinical result, especially for liposomal drugs.

**Graphical Abstract:**

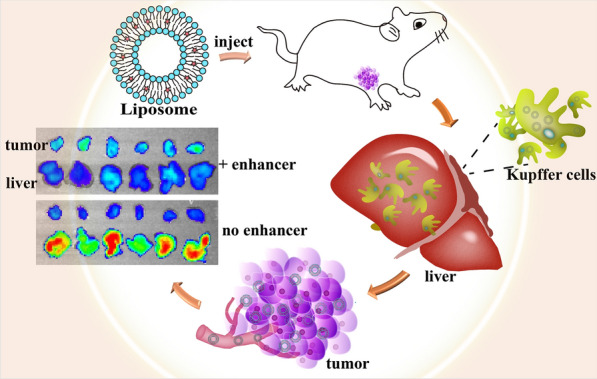

**Supplementary Information:**

The online version contains supplementary material available at 10.1186/s12951-022-01349-1.

## Background

Nearly all chemotherapeutics are cytotoxic [1–3]; they kill both tumor cells and normal cells indiscriminately in vivo, which inevitably leads to various side effects during tumor treatment [4–6]. The delivery of as many drugs as possible to solid tumors at the same dose is the key to solving this problem. As delivery vehicles [7], nanoparticles not only provide a variety of delivery systems for many bioactive insoluble drugs [8, 9] but can also protect the active drugs from degradation during blood circulation [10] to guarantee that more drug molecules are delivered to the targeted site [11–13]. Therefore, nanoparticles for tumor targeting have attracted the attention of many scholars [14–17]. In recent decades, both passive and active targeting strategies have been widely employed to achieve this aim. Drug-loaded nanoparticles in blood circulation can penetrate into tumor tissue through the gaps in the endothelial lining of blood capillaries, making use of the so-called enhanced permeability and retention (EPR) effect [18–20]. The further modification of nanoparticles with tumor targeting ligands, such as antibody [21–23], transferrin [24, 25], peptides and hyaluronic acid [26–28], on their surface, in many cases, could enhance the antitumor efficacy through specific receptor–ligand recognition and subsequent endocytosis.

Although active targeting nanoparticles are engineered to be increasingly functional and complicated, the tumor drug delivery efficiency is far from satisfactory [29–31]. A meta-analysis showed that only 0.7% (median) of the intravenously administered nanoparticles reached the tumor [32]. Dai et al. prepared trastuzumab-modified gold nanoparticles to target tumor cells with ErbB2 receptors on the surface. The data showed that less than 14 of the 1,000,000 nanoparticles interacted with tumor cells, while up to 90% of the nanoparticles were sequestered by tumor-associated macrophages (TAMs) [33]. Whatever efforts and strategies have been made, most intravenously administered nanoparticles (up to ~ 80%) still fail to avoid being cleared by the mononuclear phagocyte system (MPS) [34], and only a very small portion of nanoparticles can reach the tumor through blood circulation.

Recently, Ouyang et al. [35] discovered a simple way to effectively improve the tumor delivery and therapeutic efficacy of nanomedicine by reaching a nanomedicine threshold dose. They proved that nanoparticle clearance in vivo mainly depends on the number of receptors and binding sites available on liver Kupffer cells. A single dose with a sufficient number of nanoparticles saturating the effective binding site threshold can overwhelm the Kupffer cells, therefore reducing liver clearance, prolonging the circulation of nanoparticles and unprecedentedly enhancing the tumor delivery efficiency (up to 12%). They found that the threshold dose was more than 1 trillion nanoparticles in 24 h for mice. In this way, the tumor volume of mice in the over-threshold group was reduced by 57% and their survival time was extended by 29% in comparison with Caelyx alone in 4T1 tumor-bearing mice.

This research work laid the foundation for the threshold dose concept of more than 1 trillion nanoparticles and provided a powerful yet simple tumor drug delivery strategy. Since we did observe the phenomenon that a higher dose of nanoparticles led to higher tumor accumulation in mice (unpublished data), we hope to examine whether this over-threshold dosing strategy could unexpectedly improve the in vivo antitumor efficacy in the case of another chemotherapeutic.

**Scheme 1.**
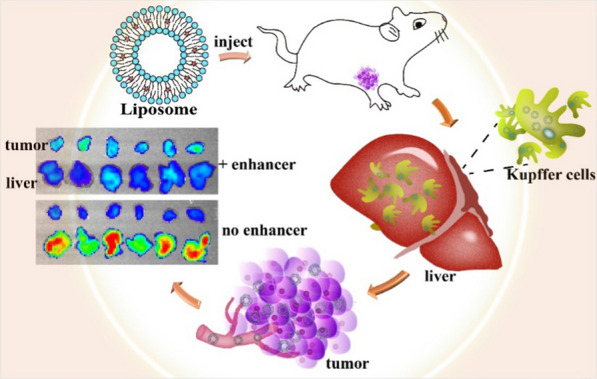
Schematic illustration of over-threshold dosing used for overwhelming Kupffer cells and enhancing tumor accumulation

Annonaceous acetogenins (ACGs), an active fraction extracted from Annona Squamosa seeds, have strong antitumor activity against various tumors, which has been verified by many studies in vitro and in vivo [36–40]. ACGs are composed of several compounds with very similar chemical structures, each made up of a long aliphatic chain (containing 34–37 carbons) bearing an α,β-unsaturated γ-lactone ring and 0–3 tetrahydrofuran (THF) ring(s) [41] (see Additional file 1: Fig. S1). However, the serious side effects and narrow therapeutic window limit ACGs application in the clinic; thus, there is an urgent need for a tumor-targeting drug delivery strategy to solve this issue. In this paper, blank PEGylated liposomes (PEG-Lipo) were used as delivery enhancers in an attempt to overwhelm Kupffer cells and enhance the tumor delivery of ACGs-loaded PEGylated liposomes (PEG-ACGs-Lipo) based upon the theory of over-dose threshold drug delivery (Scheme 1).

## Materials and methods

### Materials

Soybean phospholipids (SPC) were purchased from Shenyang Tianfeng Biological Pharmaceutical Co., Ltd. (Shenyang, China). DSPE-mPEG2000 was supplied by Shanghai ToYongBio Tech. Inc. (Shanghai, China). Cholesterol was provided by Shanghai Yuanye Bio-Technology Co., Ltd. (Shanghai, China). ACGs were provided by Professor Jianyong Si's laboratory (Institute of Medicinal Plant Development [IMPLAD], Beijing, China). 1,1-dioctadecyl-3,3,3,3-tetramethylindotricarbocyanine iodide (DiR) was purchased from AAT Bioquest Inc. (Sunnyvale, USA). Paclitaxel (PTX) injections were supplied by Beijing Union Pharm Ltd. (Beijing, China). Temozolomide (TMZ) was obtained from Dalian Meilun Biological Technology Co. Ltd. (Dalian, China). All the other reagents were of analytical grade or higher. Deionized water was used in the experiments.

### Cell lines and animals

The 4T1 and U87 MG cell lines were purchased from China infrastructure of cell line resource. 4T1 cells were cultured in RPMI 1640 medium, and U87 MG cells were maintained in MEM medium at 37 ℃ with 5% CO_2_ (Thermo311, Waltham, MA, USA). The media were supplemented with 10% fetal calf serum (FBS) and 100 U/mL penicillin and streptomycin (Gibco, St Louis, MO, USA).

Female BAL B/c mice (20 ± 2 g, 6–8 weeks old) and female BAL B/c nude mice (20 ± 2 g, 6–8 weeks old) were provided by Vital River Laboratory Animal Technology Co., Ltd. (Beijing, China). All mice were kept under a 12 h light–dark cycle environment with a relative humidity of 70 ± 5% at 25 ℃. All animal experiments were conducted in accordance with the Guidelines for Ethical and Regulatory for Animal Experiments as defined by the Institute of Medicinal Plant Development (IMPLAD), China. Ethical approval for this study was granted by the ethics committee of IMPLAD.

### Preparation of liposomes

Liposomes were prepared according to the ethanol infusion method. The specific procedure was as follows: a mixture of SPC, DSPE-mPEG2000, cholesterol, and ACGs at a weight ratio of 24:6:5:2 was co-dissolved in 1 mL ethanol and then slowly dropped into deionized water under ultrasonication (250 W, 25 ℃ ± 2 ℃, Kun Shan Ultrasonic Instruments Co., Ltd, Kunshan, PR China). The ethanol was removed by evaporation under reduced pressure at 45 ℃ to obtain PEG-ACGs-Lipo. When ACGs were absent in the formulation, the same procedure was used to form PEG-Lipo. The low drug loading content of PEG-ACGs-Lipo (LDLC PEG-ACGs-Lipo) was also fabricated at a weight ratio of 53:13.3:11:0.2 (SPC:DSPE-mPEG2000:cholesterol:ACGs) to dose the mice with the required dose of ACGs and an over-threshold of nanoparticles.

DiR, a near-red fluorescent dye [42–44], was used as a probe to trace the in vivo distribution of liposomes. DiR-labeled PEG-ACGs-Lipo were prepared by the addition of DiR to the mixture following the same procedure.

### HPLC analysis of ACGs

Squamocin, the major compound with the highest content in ACGs, was used as the quantitative index of ACGs. The accurate determination of ACGs concentration is determined by HPLC system (DIONEX Ultimate 3000, USA) using a UV detector (208 nm) and Venusil XBP C18 (L) column (4.6 mm × 250 mm, 5 μm; Agela, China) with the mobile phase consisted of 0.3% phosphoric acid solution and acetonitrile (3:7, v/v). The flow rate was 1.0 mL/min.

### Characterization of liposomes

A dynamic light scattering instrument (DLS; Zetasizer Nano ZS, Malvern Instruments, UK) was used to detect the mean particle size, polydispersity index (PDI), and zeta potential of PEG-ACGs-Lipo and PEG-Lipo at 25 ℃. Each measurement was performed in triplicate with 12 runs.

The structure and morphology of liposomes were observed by transmission electron microscopy (TEM, JEM-1400, Tokyo, Japan). Briefly, 6.0 μL of water-diluted liposomes was dropped on a 300-mesh copper net, allowed to stand for 5 min, stained with 2% (w/v) uranyl acetate liquid for 90 s, air dried, and then observed under TEM at an accelerating voltage of 120 kV.

The lyophilized powder of PEG-ACGs-Lipo and LDLC PEG-ACGs-Lipo was dissolved in methanol and centrifuged at 13,000 rpm for 10 min to completely release ACGs from the liposomes. The supernatant was analyzed by HPLC to determine the total ACGs content in lyophilized powder. Liposomes (0.4 mL) was put into an Ultra centrifugal filter (0.5 mL, NMWL 10 k, Millipore, USA) and centrifuged at 13,000 rpm for 30 min. The filtrate was analyzed by HPLC for free ACGs content. The drug loading content (DLC) and encapsulation efficiency (EE) were calculated according to the following formula:

1 $$\mathrm{DLC }\left(\mathrm{\%}\right)=\frac{{\mathrm{W}}_{\mathrm{a}}}{{\mathrm{W}}_{\mathrm{l}}}\times 100\mathrm{\%},$$

2 $$\mathrm{EE }\left(\mathrm{\%}\right)=\frac{{\mathrm{W}}_{\mathrm{t}}-{\mathrm{W}}_{\mathrm{f}}}{{\mathrm{W}}_{\mathrm{t}}}\times 100\mathrm{\%},$$

where W_a_ and W_l_ are the total weight of ACGs in lyophilized powder and the weight of the lyophilized powder, respectively, and W_f_ and W_t_ are the weight of free ACGs and the weight of total ACGs in liposomes, respectively.

### The stability of liposomes in physiological media and plasma

PEG-ACGs-Lipo or PEG-Lipo were mixed with 1.8% NaCl solution and 10% glucose solution, respectively, at a volume ratio of 1:1 or mixed with PBS, artificial gastric juice, artificial intestinal juice and plasma, respectively, at a volume ratio of 1:4, followed by incubation at 37 ℃. The particle size of the mixture was measured at different time intervals, and the possible physical changes, such as turbidity and precipitation, were also monitored to examine the stability of PEG-ACGs-Lipo or PEG-Lipo in physiological media and plasma. Each experiment was performed in triplicate.

### The effect of dose on the tumor delivery of PEG-liposomes in 4T1 tumor-bearing mice

4T1 cells in logarithmic phase were suspended in RPMI medium without FBS, and 2.0 × 10^6^ cells were subcutaneously inoculated into the right armpit of female BAL B/c mice to establish a 4T1 tumor-bearing mouse model. Once the tumor size reached ~ 500 mm^3^, the mice were randomly divided into three groups. Three doses of 0.2 mL of PEG-Lipo (12 mg/kg, 50 mg/kg, 80 mg/kg, calculated by phospholipids) were injected intravenously. Then, the mice were imaged using IVIS Living Image@ 4.4 (Caliper Life Sciences, Hopkinton, MA, USA) at different time intervals (745 nm/800 nm excitation/emission filters). The detailed parameters for imaging were as follows: a binning of 8; automatic exposure time, f/stop of 2; subject height of 1.5 cm; and field of view of 25 cm (in vivo) or 14 cm (ex vivo). At the end of the experiment, all the mice were sacrificed, and their tumors and major organs were excised and imaged as described above. Living Image software (version 4.2) was used for quantitative analysis. The relative tumor targeting index (RTTI) was calculated according to the following formula:

3 $$\mathrm{RTTI}=\frac{{\mathrm{ROI}}_{\mathrm{t}}}{{\mathrm{ROI}}_{\mathrm{l}}}$$

where ROI_t_ and ROI_l_ are the average fluorescence intensity of the tumor and liver, respectively.

### In vitro cytotoxicity assay of liposomes

4T1 cells and U87 MG cells in the logarithmic phase were seeded in 96-well plates (5000 cells/well) and incubated for 24 h at 37 °C with 5% CO_2_. Then, the culture medium was replaced by a series of concentrations (calculated by ACGs) of PEG-ACGs-Lipo (100 μL) or free ACGs (100 μL) with blank medium or 0.5% DMSO solutions as a negative control, followed by incubation for 72 h. Afterward, 20 μL MTS (Promega, USA) was added to each well and incubated for 3 h. The absorbance of the sample plates was then measured using a plate reader (Biotek Synergy H1, VT, USA) at 490 nm. The cell survival rate was calculated according to the following formula:

4 $$\mathrm{Cell viability rate }\left(\mathrm{\%}\right)=\frac{{\mathrm{OD}}_{\mathrm{t}}}{{\mathrm{OD}}_{n}}\times 100\mathrm{\%},$$

where ODt and ODn are the mean optical densities of the treated group and negative control group, respectively.

The 50% inhibitory concentration value (IC50) was calculated using GraphPad Prism Software, Version 5 (GraphPad Software, Inc., La Jolla, CA, USA).

### In vivo antitumor efficacy and biodistribution by over-threshold dosing in 4T1 tumor-bearing mice

A 4T1 tumor-bearing mouse model was established as described above. When the tumor volume reached ~ 100 mm^3^, the mice were randomly divided into 5 groups (n = 6): the negative control group (normal saline), positive control group (PTX injection; 8 mg/kg), PEG-ACGs-Lipo group (0.15 mg/kg equivalent ACGs, corresponding to 2.25 mg/kg of phospholipids), PEG-Lipo + PEG-ACGs-Lipo group (50 mg/kg equivalent phospholipids of blank PEGylated liposomes as enhancer, plus 0.15 mg/kg equivalent ACGs of PEG-ACGs-Lipo), and low drug loading content (LDLC) of PEG-ACGs-Lipo group (0.15 mg/kg equivalent ACGs, corresponding to 50 mg/kg of phospholipids). A total of 0.2 mL of the corresponding drug was injected intravenously every other day for 7 doses. Tumor size and body weight were measured every two days. Tumor volume was calculated using Formula (5). Twenty-four hours after the final dose, the mice were sacrificed according to institutional guidelines, and the tumors were dissected and weighed. The tumor inhibition rate (TIR) was calculated using Eq. (6):

5 $$\mathrm{V}\left({mm}^{3}\right)=0.5{\mathrm{ab}}^{2} {mm}^{3},$$

where a is the longest diameter of the tumor, and b is the shortest diameter of the tumor.

6 $$\mathrm{TIR}\left(\mathrm{\%}\right)=\left(1-\frac{{\mathrm{W}}_{\mathrm{t}}}{{\mathrm{W}}_{\mathrm{n}}}\right)\times 100\mathrm{\%},$$

where Wt and Wn are the mean tumor weights of the treated group and negative control group, respectively.

To compare the effects of below-threshold dosing and over-threshold dosing on the in vivo biodistribution of ACGs, DiR-labeled PEG-ACGs-Lipo was used at the final dose. The mice were sacrificed by cervical dislocation 24 h later, and the tumor, heart, liver, spleen, lung, kidney, and brain were dissected and imaged as described above.

### In vivo antitumor efficacy and biodistribution in U87 MG tumor-bearing mice

U87 MG cell suspensions (0.2 mL, 2.5 × 10^7^ cells/mL) were inoculated subcutaneously into the right armpit of female BAL B/c nude mice. When the tumor size reached 1000 mm^3^, the tumors were dissected and cut into 2 mm × 2 mm × 2 mm pieces and then transplanted into the right armpit of mice by an inoculation needle to establish a U87 MG tumor-bearing mouse model. When the tumor volume reached ~ 100 mm^3^, the mice were divided into 4 groups (n = 6): the negative control group (normal saline), positive control group (25 mg/kg of TMZ), PEG-ACGs-Lipo group (0.2 mg/kg equivalent ACGs), and PEG-Lipo (80 mg/kg of phospholipids) + PEG-ACGs-Lipo group (0.2 mg/kg equivalent ACGs). The positive group was administered orally every day for a total of 15 times, and the other groups were administered 8 times intravenously every other day. The subsequent operations are as described above, except that TIR was calculated according to Formula (7).

7 $$\mathrm{TIR}\left(\mathrm{\%}\right)=\left(1-\frac{{\mathrm{V}}_{\mathrm{t}}}{{\mathrm{V}}_{\mathrm{n}}}\right)\times 100\mathrm{\%},$$

where Vt and Vn are the mean tumor volumes of the treated group and negative control group, respectively.

### Statistical analysis

The statistical analysis among the different groups was conducted using IBM SPSS Statistics software, Version 19 (IBM Corporation, Armonk, NY, USA). P < 0.05 was considered statistically significant.

## Results and discussion

It has been indicated in Ouyang’s work that different types of nanoparticles, due to their different surface properties, absorb different plasma proteins and then form different protein profiles and are thus taken up by different types of Kupffer cells via receptor-mediated phagocytosis. Kupffer cells comprise a heterogeneous and powerful population, and each cell clears a specific nanoparticle type. Therefore, over-threshold co-dosing can improve the tumor delivery of only the same type of nanoparticles; for example, the co-injection of large dose of blank liposomes can improve the tumor delivery for liposomes only, but not for gold nanoparticles.

Since PEGylated liposomes have been extensively investigated in 4T1 tumor-bearing mice in Ouyang’s work, we used PEGylated liposomes to load ACGs and tried to improve the tumor accumulation and antitumor efficacy of ACGs by means of the co-injection of over-threshold of blank liposomes [35].

### Preparation and characterization of liposomes

**Fig. 1:**
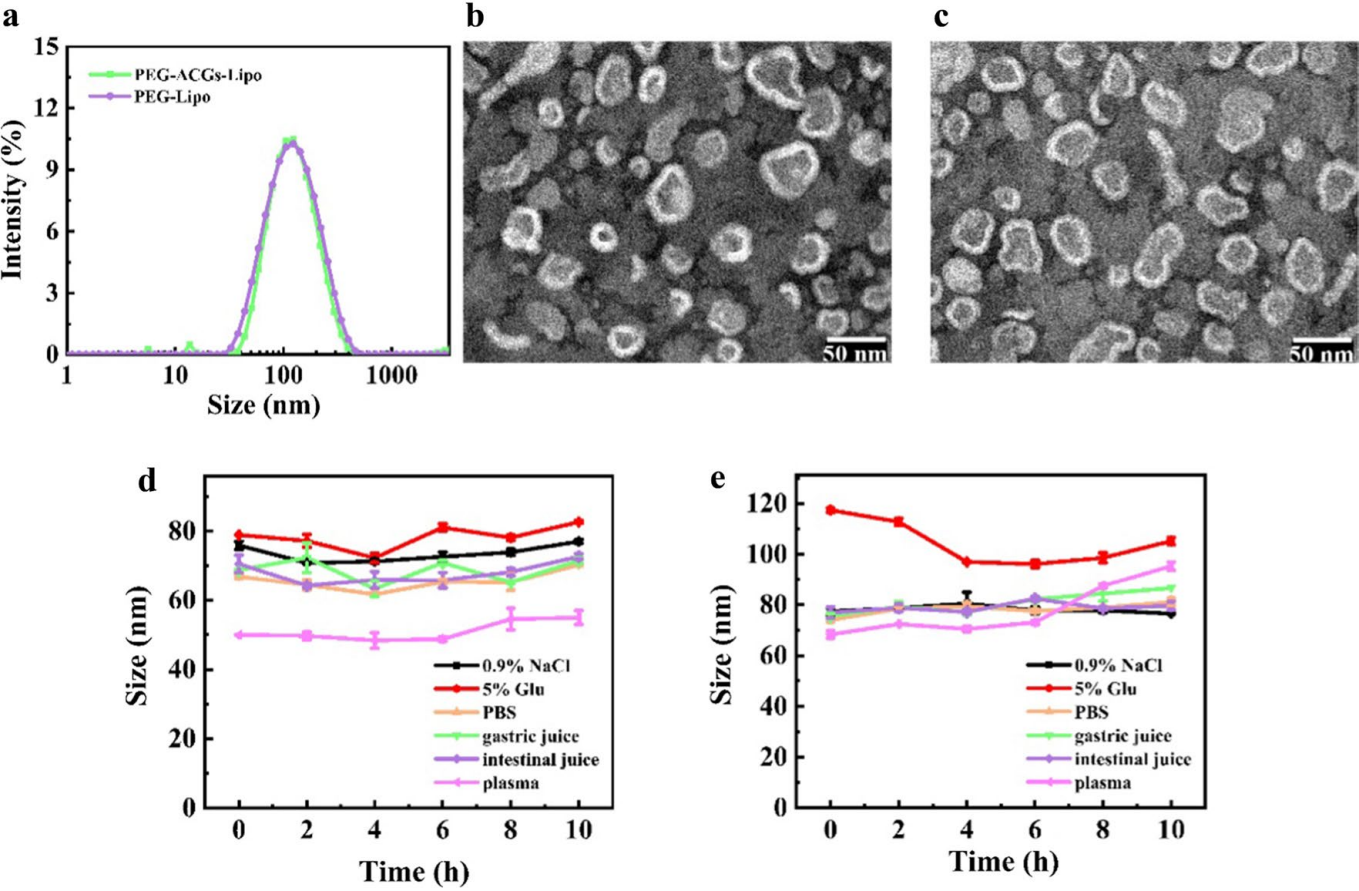
Characterization and their stability in physiological media of two liposomes. **a** The particle size distribution of the two liposomes. The morphology of **b** PEG-ACGs-Lipo and **c** PEG-Lipo. The particle size change curve of **d** PEG-Lipo and **e** PEG-ACGs-Lipo in physiological media

Liposomes can be prepared by a number of methods. We tried to fabricate ACGs-loaded liposomes through thin-film sonication and ethanol injection methods and found that the latter was more suitable for encapsulating ACGs into liposomes with smaller particle sizes and higher encapsulation efficiency at a wide range of phospholipid concentrations than the former. As shown in Fig. 1a, the resultant PEG-Lipo had a particle size of 85.6 ± 0.6 nm, a narrow particle size distribution (PDI, 0.31 ± 0.01) and a negative zeta potential of − 23.6 ± 0.8 mV, while PEG-ACGs-Lipo displayed a similar DLS parameter with a particle size of 82.4 ± 0.0 nm, a PDI value of 0.31 ± 0.01, and a zeta potential of - 24.0 ± 1.2 mV. The DLC and EE of PEG-ACGs-Lipo were 3.9 ± 0.6% and 97.1 ± 0.5%, respectively. Both PEG-ACGs-Lipo and PEG-Lipo exhibited a near-spherical shape with a typical bilayer structure under TEM (Fig. 1b, c).

The content of ACGs in the formulation of PEG-ACGs-Lipo was very low (3.9%), and ACGs, due to their lipophilic property, were located in the phospholipid bilayer of liposomes, so the loading of ACGs in PEG-Lipo was supposed to have little effect on the surface property of the resultant liposomes. Thus, PEG-Lipo and PEG-ACGs-Lipo may have similar in vivo behavior.

After DiR labeling, PEG-Lipo and PEG-ACGs-Lipo showed little augmentation in their average particle size and shape (Additional file 1: Fig. S2). We also made LDLC PEG-ACGs-Lipo at a weight ratio of 53.0:13.3:11.0:0.2 (SPC:DSPE-mPEG2000:cholesterol:ACGs) to meet the requirement of over-threshold nanoparticles when intravenously administered and maintain the same ACGs dose without the need for blank liposomes as an enhancer. Not surprisingly, LDLC PEG-ACGs-Lipo displayed a much lower DLC of 0.19 ± 0.00% but a slightly higher EE of 98.1 ± 0.6% than PEG-ACGs-Lipo. We expect it to achieve the same effect as PEG-ACGs-Lipo + blank PEG- Lipo.

### The stability of liposomes in physiological media

Here, the stability of liposomes in physiological medium and in plasma was investigated. As seen in Fig. 1d, e, both PEG-ACGs-Lipo and PEG-Lipo were quite stable in normal saline, 5% glucose, PBS, artificial gastric juice, artificial intestinal juice or plasma with limited particle size change, which demonstrates their suitability for either oral administration or intravenous injection.

### The effect of dose on tumor delivery of PEG-liposomes in 4T1 tumor-bearing mice

In Ouyang’s work, the calculation of the number of liposomes and gold nanoparticles was based on theoretical data and certain assumptions. The calculation was complicated, and the result was also an estimated value. To conveniently evaluate the particle number of PEG-Liposomes in this study, we tried to use the dose of phospholipid as an indicator of the particle number of liposomes with similar mean particle sizes and PDI values. In Ouyang’s work, Caelyx liposomes that were commercially available (dose of 2 mg/kg) contained 4.6 trillion liposomal nanoparticles. Since a dose of 2 mg/kg of Caelyx liposomes contains 9.58 mg/kg HSPC, 3.19 mg/kg cholesterol, and 3.19 mg/kg DSPE-mPEG, we selected 2.25 mg/kg of phospholipids (SPC + DSPE-mPEG) of PEG-ACGs-Lipo (the dose of 0.15 mg/kg ACGs) as a below-threshold dose and selected 50 mg/kg and 80 mg/kg of phospholipids of PEG-Lipo as enhancers to augment the particle number dose of PEG-Lipo, reduce liver accumulation and increase tumor delivery. The resultant PEG-ACGs-Lipo showed a particle size (∼ 82.4 nm) that was similar to that of the Caelyx liposomes (∼ 86.9 nm) used in Ouyang’s work. The resultant PEG-Lipo displayed a particle size (∼ 85.6 nm) that was also similar to that of Caelyx-similar liposomes without the doxorubicin (∼ 102.2 nm) used as enhancers in Ouyang’s work. However, for different types of nanoparticle, such as micelles, microemulsions, and polymeric nanoparticles, the relationship between vehicle weight and corresponding particle number needs to be standardized according to the composition of nanoparticles and calibrated according to complex calculation. Here, the effect of phospholipid dose on the tumor delivery of PEG-liposomes in 4T1 tumor-bearing mice was investigated.

**Fig. 2:**
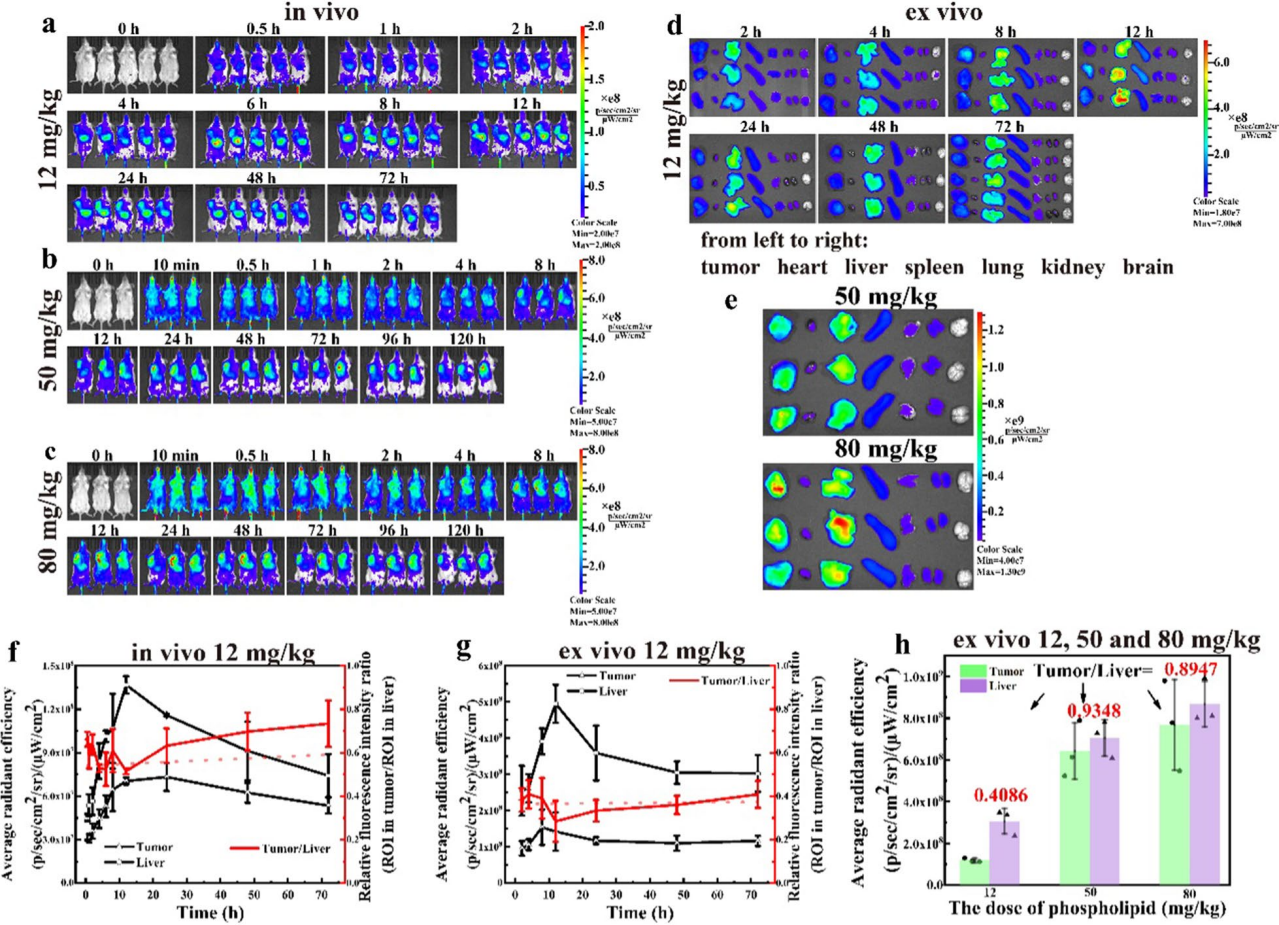
The effect of dose on tumor delivery of PEG-liposomes in 4T1 tumor-bearing mice. The dynamic biodistribution of PEG-Lipo via whole-mouse imaging at doses of **a** 12 mg/kg, **b** 50 mg/kg and **c** 80 mg/kg equivalent phospholipids. The ex vivo distribution of PEG-Lipo at doses of **d** 12 mg/kg and **e** 50 mg/kg and 80 mg/kg equivalent phospholipids. At a dose of 12 mg/kg, the half-quantitative analysis of the fluorescence intensity in tumor and liver **f** in vivo and **g** ex vivo. **h** The ex vivo fluorescence intensity in the tumor and liver at the end of the experiment. Data represent the mean ± SD (n = 3)

As seen in Fig. 2a, f, after the intravenous administration of 12 mg/kg of DiR-labeled PEG-Lipo, fluorescence quickly accumulated in the liver of mice, slightly concentrated in the liver at 0.5 h, significantly concentrated at the 1st hour, peaked at the 12th hour, then slowly declined. Obvious fluorescence accumulation in tumors was observed from the 2nd hour post dose and then slowly increased, peaked at the 24th hour, and then slowly declined. At all timepoints throughout the 72 h of observation, the fluorescence intensity in the liver was significantly stronger than that in the tumor, indicating that at this dose, the injected PEG-Lipo was mainly cleared by liver Kupffer cells and seldom delivered to the tumor. In this case, the uptake of liposomes by liver Kupffer cells was far from saturation.

To verify the feasibility of the dynamic fluorescence distribution in whole mice, three mice were euthanized at specific timepoints, and their tumor and major organs were dissected for direct imaging. This indicated that the actual fluorescence intensity in the dissected liver and tumor at different time intervals (Fig. 2d) was well correlated with the dynamic fluorescence profile in the liver and tumor (Fig. 2a) on the basis of whole -mouse imaging. Figure 2d more distinctly demonstrated that PEG-Lipo was mainly distributed in the liver, less in the tumor and spleen, even less in the lung and least in the brain. The quantitative profiling of liver fluorescence and tumor fluorescence in vivo (Fig. 2f) and ex vivo (Fig. 2g) depicted the targeting efficiency change over time. Due to differences in the fluorescence intensity detected from the superficial (such as tumor) and deep (such as liver) locations of the mouse body during in vivo whole animal imaging, the liver fluorescence measured was usually significantly weaker than it truly was, and the tumor fluorescence measured was relatively close to the actual intensity. This is why the RTTI (~ 0.58, the midpoint of the dotted fitting line in Fig. 2f) was higher than that ex vivo (~ 0.38, the midpoint of the dotted fitting line in Fig. 2g), and the latter was no doubt more accurate. It was also clear that whatever ex vivo or in vivo, the targeting efficiency was relatively stable 24 h post dose, probably due to the complete biodistribution of the dosed nanoparticles within the 12–24th hours, followed by similar deletion in the liver and tumor. Therefore, it was reasonable to select the actual observed biodistribution in tumors versus in the liver at the same timepoint post dose to compare the tumor targeting efficiency among different drug delivery systems.

When the dose was increased to 50 mg/kg or 80 mg/kg of phospholipids, the dynamic fluorescence distribution of PEG-Lipo was quite different. As shown in Fig. 2b, liver fluorescence was observed 10 min post dose but was maintained at a much lower level throughout the whole period of observation until 120 h. The fluorescence in the liver became very weak at 48th hour and then weakened. In contrast, tumor fluorescence accumulation occurred as early as 0.5 h post dose, continuously increased with a significant increase at the 8th hour, and maintained a plateau level from the 24th hour until the 120th hour. Even at the 120th hour, the fluorescence in the tumor was still very strong. This meant that when dosed at 50 mg/kg phospholipids, PEG-Lipo could quickly and effectively saturate Kupffer cell uptake, therefore allowing more PEG-Lipo to accumulate in tumors through blood circulation. Our work not only verified the improved tumor accumulation by the over-threshold dosing of nanoparticles but also found over-threshold dosing could significantly advance the time that nanoparticles start to accumulate in tumors from the circulation, leading to a higher and quicker accumulation in tumors and a lower accumulation in the liver.

In the case of 80 mg/kg phospholipids (Fig. 2c), the dynamic fluorescence distribution of PEG-Lipo was similar. This meant that when dosed over the threshold, a further increase in the dose may not elevate the tumor targeting efficiency but can accelerate the distribution of dosed nanoparticles to the tumor. At the end of the experiment (Fig. 2e), the two doses reached a similar RTTI of ~ 0.9 (Fig. 2h, 0.9358 for 50 mg/kg, 0.8947 for 80 mg/kg), about 2.25-fold in contrast to that in case of 12 mg/kg group (0.4086).

Overall, this part of the work showed that overthreshold dosing not only elevates the tumor targeting efficiency (from ~ 0.4 to ~ 0.9) but also accelerates the distribution of dosed nanoparticles to tumors. In Ouyang’s work, although a high dose successfully enabled up to 12% of injected nanoparticles to reach the tumor, the tumor targeting efficiency (tumor drug/liver drug) was not satisfactory, and was less than 1 in nearly all of the cases. This means that there is still a long way to go, and more strategies need to be integrated to further enhance the tumor delivery of chemotherapeutics. To compare the therapeutic effects of different doses, the antitumor efficacy in vivo was investigated with and without enhancers.

### In vitro cytotoxicity assay of liposomes

**Fig. 3:**
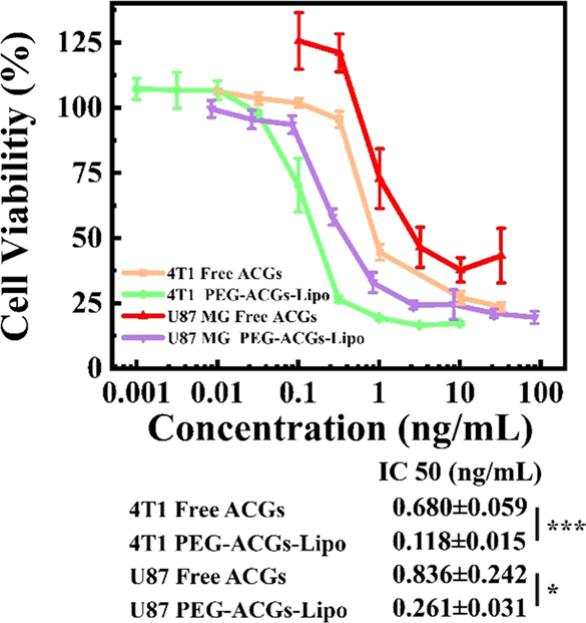
The proliferation inhibitory profile of free ACGs and PEG-ACGs-Lipo against 4T1 cells and U87 MG cells. ^***^P < 0.001, ^**^P < 0.05 vs. Free ACGs. Data represent the mean ± SD (n = 3)

The in vitro cytotoxicity of the ACGs solution and PEG-ACGs-Lipo against 4T1 cells and U87 MG cells was investigated by MTS test. As shown in Fig. 3, the cell viability of 4T1 cells and U87 MG cells showed a dose-dependent manner after incubation with ACGs solution and PEG-ACGs-Lipo for 72 h. Free ACGs showed strong cytotoxicity to 4T1 cells and U87 MG cells, with IC50 values of 0.680 ± 0.059 ng/mL and 0.836 ± 0.242 ng/mL, respectively, while PEG-ACGs-Lipo displayed stronger proliferation inhibitory activity, with IC50 values of 0.118 ± 0.015 ng/mL (for 4T1) and 0.261 ± 0.031 ng/mL (for U87 MG), respectively. The reason is probably that encapsulation into liposomes enhanced the cellular uptake of ACGs by tumor cells, as is often reported in nanomedicine [45–47].

### In vivo antitumor efficacy and biodistribution in 4T1 tumor-bearing mice

**Fig. 4:**
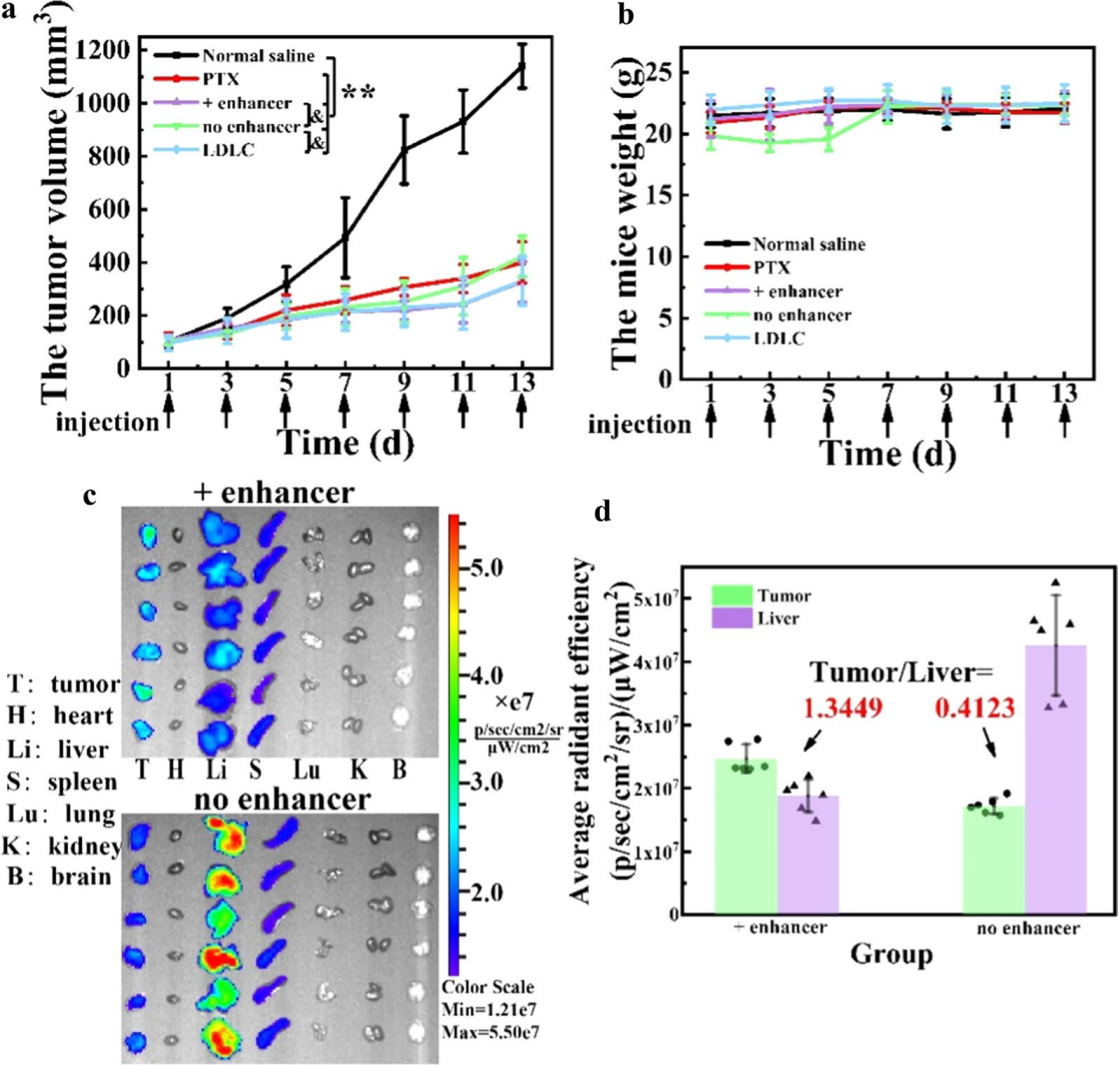
In vivo antitumor efficacy and biodistribution in 4T1 tumor-bearing mice. **a** During the experiment, the tumor growth curve of mice in the four groups was delineated. ^**^P < 0.01 vs. Normal saline; ^&^*P* < 0.05 vs. no enhancer. **b** The weight change of mice with time in each group. **c** At 24 h after administration, the distribution of the + enhancer group and the no enhancer group in related tissues and organs of mice was imaged. **d** At 24 h after administration, the relative fluorescence intensity and ratio of the + enhancer group and the no enhancer group in the tumor and liver were calculated. Data represent the mean ± SD (n = 6)

To verify whether the over-threshold dosing can bring significant and practical beneficial effects in tumor treatment, we selected blank PEG-Lipo (50 mg/kg equivalent phospholipids) as a delivery enhancer to examine whether a co-injection of this enhancer could significantly improve the antitumor efficacy of PEG-ACGs-Lipo alone (0.15 mg/kg equivalent ACGs, 2.25 mg/kg equivalent phospholipids). We also fabricated LDLC PEG-ACGs-Lipo containing 0.15 mg/kg equivalent ACGs and 50 mg/kg equivalent phospholipids to examine whether they could achieve improved antitumor therapeutic efficacy similar to that of PEG-ACGs-Lipo + enhancer. PTX injection (8 mg/kg) was employed as the positive control. As shown in Fig. 4a, a rapid growth of tumor volume was observed in the negative control group, while the tumors in the other three groups grew much slower, especially the co-injection group (PEG-ACGs-Lipo + enhancer), in which the tumor volume of the mice was reduced by 22.6% (vs. PEG-ACGs-Lipo alone, *P* < 0.05). Meanwhile, we found that LDLC PEG-ACGs-Lipo displayed a very close tumor volume growth curve (Fig. 4a).

At the end of the experiment, the tumor was dissected and weighed, and the TIR was calculated (Table 1). The PEG-ACGs-Lipo alone (no enhancer group) showed slightly lower tumor-suppressive effects than the positive group (TIR, 57.21% vs. 59.04%), while the + enhancer group displayed significantly higher TIR than the no enhancer group (65.82% vs. 57.21%, *P* < 0.05). Likewise, LDLC PEG-ACGs-Lipo achieved a TIR (67.58%) similar to that of the + enhancer group. All the groups showed similar body weight change profiles except for a small drop in the initial days followed by recovery for the PEG-ACGs-Lipo group (Fig. 4b).

**Table 1:** In vivo antitumor efficacy against 4T1 tumor-bearing mice

Group	Tumor weight (g)	Tumor inhibition rate (%)
Normal saline	1.27 ± 0.14	NA
PTX	0.52 ± 0.11^**^	59.04 ± 8.65
+ enhancer	0.44 ± 0.04^**&^	65.82 ± 2.93^&^
no enhancer	0.54 ± 0.08^**^	57.21 ± 6.29
LDLC	0.41 ± 0.14^**&^	67.58 ± 10.64^&^

Results are presented as mean ± SD (n = 6).

^**^P < 0.01 vs. Normal saline; ^&^*P* < 0.05 vs. no enhancer

The ex vivo fluorescence imaging of tumors and major organs dissected 24 h after the last dose visualized the biodistribution of PEG-ACGs-Lipo with or without the enhancer (Fig. 4c). It was clear that when dosed alone and below the threshold, PEG-ACGs-Lipo was mainly distributed in the liver, with an RTTI of only 0.4123. However, with the help of co-injected blank liposomes (+ enhancer), PEG-ACGs-Lipo preferentially moved to the tumor, and the RTTI reached 1.3449 (Fig. 4d) at the 24th hour post dose, with the liver fluorescence intensity reduced by 56% and the tumor fluorescence intensity increased by 43%, once again demonstrating the excellent tumor delivery enhancement of a large dose of blank liposomes. It also supported that when the dose was 50 mg/kg, the number of PEG-Lipo was enough to saturate Kupffer cells and reduce the uptake of the reticuloendothelial system in the liver.

The RTTI (0.4123) of the multidosed PEG-ACGs-Lipo (below-threshold) was quite consistent with that of the single dose (12 mg/kg), while in the case of over- threshold dosing, the multidosed PEG-ACGs-Lipo + enhancer (over-threshold) led to significantly enhanced targeted efficiency (1.3449) compared with that of the single dose (0.9348), probably because the delivery-enhancing effect of over-threshold administration could be to some extent accumulated by multiple doses.

Slightly surprisingly, this significantly improved tumor targeting efficiency brought a limited elevation in TIR of PEG-ACG-Lipo (< 10%), which was much less than that achieved in Ouyang’s work (57% reduction in tumor volume and 29% extension in survival time). One reason may be that Caelyx alone at the given dose reached a lower TIR (38% vs. 57% in our study). Another reason may be that in comparison with DOX, ACGs are hydrophobic and less able to penetrate into the depth of the tumor or are less taken up by tumor cells. It was also reported that PEGylated liposomes usually easily accumulated at the blood capillary near the tumor, but it was difficult for them to penetrate capillary walls and enter the tumor environment [48, 49]. As stated in many studies [50–52], good tumor accumulation is only the second step in the five cascaded steps for nanomedicine to truly exert antitumor efficacy, and presentable penetration to the depth of tumor tissue across the blood capillary would be the next crucial step.

### In vivo antitumor efficacy and biodistribution in U87 MG tumor-bearing mice

**Fig. 5:**
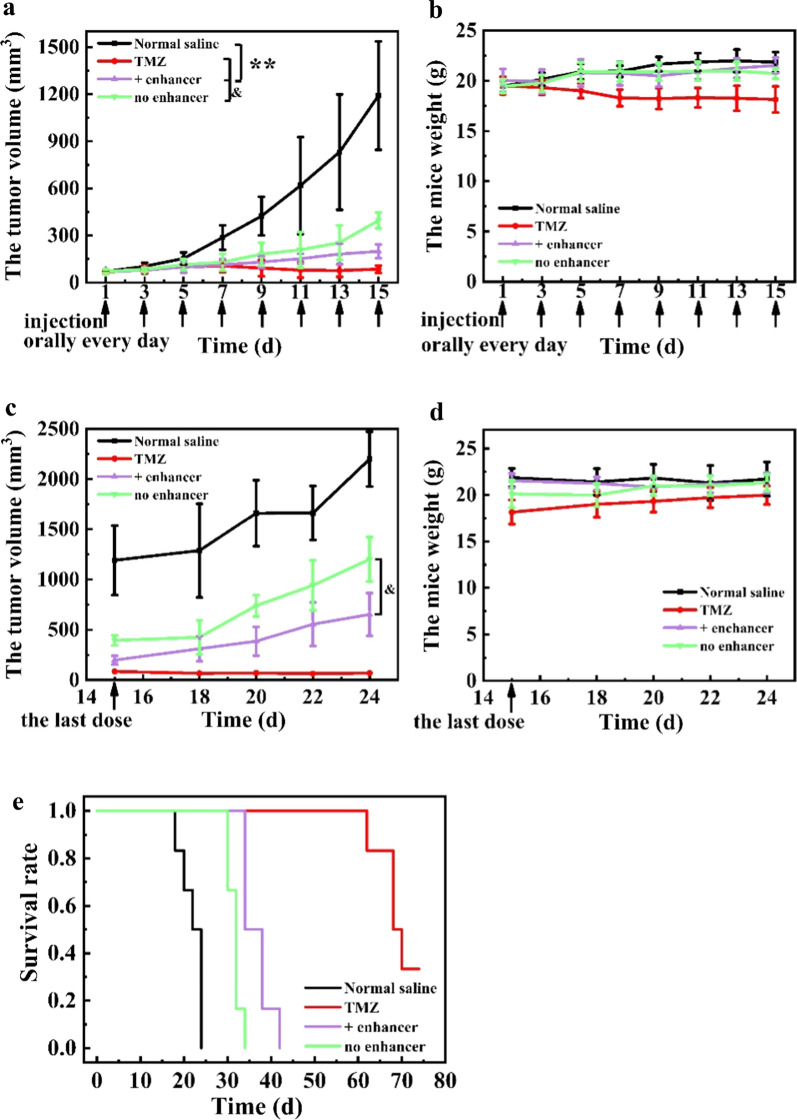
In vivo antitumor efficacy in U87 MG tumor-bearing mice. **a** The mouse tumor volume change curve over time during administration. ^**^P < 0.01 vs. Normal saline, and ^&^*P* < 0.05 vs. no enhancer. **b** The mouse weight change curve over time during administration. **c** The mouse tumor volume change curve over time after the last dose, and ^&^*P* < 0.05 vs. no enhancer. **d** The mouse weight change curve over time after the last dose. **e** Survival rate of mice in each group. Data represent the mean ± SD (n = 6)

In another mouse model, PEG-Lipo (80 mg/kg equivalent phospholipids) was used as an enhancer for co-injection to investigate its effect on tumor delivery and the antitumor efficacy of PEG-ACGs-Lipo in U87 MG tumor-bearing mice. The three groups of mice that received TMZ or PEG-ACGs-Lipo all displayed much slower tumor growth than the negative control group (P < 0.01) (Fig. 5a). Compared with PEG-ACGs-Lipo alone, co-injection of blank PEG-Lipo significantly reduced tumor size by 49.8% (P < 0.05) (Fig. 5a; Table 2), and increased the TIR from 66.80% to 83.32% (P < 0.05). It is worth mentioning that in terms of the TIR, TMZ displayed a particularly excellent antitumor effect, but the body weight of mice that received TMZ decreased continuously during the whole administration period, probably due to the high drug dose of 25 mg/kg, while the body weight of mice in the other groups slowly increased (Fig. 5b).

**Table 2:** The tumor volume and the corresponding tumor inhibition rate calculated at the 15th day post dose in U87 MG tumor-bearing mice

Group	The tumor volume (mm^3^)	Tumor inhibition rate (%)
Normal saline	1191.77 ± 345.67	NA
TMZ	85.41 ± 23.82^**^	92.83 ± 2.00
+ enhancer	198.76 ± 43.48^**^	83.32 ± 3.65^&^
no enhancer	395.61 ± 50.62^**^	66.80 ± 4.25

Results are presented as mean ± SD (n = 6).

^**^P < 0.01 vs. Normal saline; ^&^ *P* < 0.05 vs. no enhancer

After the last dose, the tumor volume and the body weight of the mice were monitored (Fig. 5c, d). In comparison with PEG-ACGs-Lipo alone, the co-injection of blank PEG-Lipo resulted into slower tumor growth (Fig. 5c) and a longer survival time (42 days vs. 32 days, Fig. 5e). All the groups displayed a slow body weight increase (Fig. 5d).

## Conclusions

In this paper, by using phospholipid dose instead of particle number as an indicator, we verified that the co-injection of a large dose of blank liposomes or direct dosing over the threshold of a low drug loading content of liposomes truly enhanced the tumor delivery and improved the antitumor efficacy of liposomal therapeutics at the same drug dose in both 4T1 and U87 tumor-bearing mouse models. Threshold dose theory, which is based on particle number and over-threshold administration strategy, provided a simple and feasible way for nanomedicine to enhance its tumor delivery and therapeutic efficacy. However, this improvement was less than expected, as tumor accumulation is just one of the five key cascaded steps for nanomedicines to fully exert their action. Meanwhile, the additional infusion of a large number of blank vehicles in blood circulation may cause side effects, such as potential immunogenicity and aggravation of the burden on the liver and kidney. Besides, in case of liposomes that contained large amount of internal aqueous phase thus allow not too much phospholipid to reach the necessary threshold of particle number, or low drug loading nanomedicine (such as nano-emulsions) that easily reach the threshold, over-threshold administration will prove to be an easy and feasible way to enhance their clinic benefit at very low cost.

## Supplementary Information

**Additional file 1: Fig. S1.** The chemical structures of **a** squamocin and **b** bullatacin (the two major components in ACGs). **Fig. S2.** Characterization of two DiR-labeled liposomes. **a** The particle size distribution of two DiR-labeled liposomes. The morphology of **b** DiR-labeled PEG-ACGs-Lipo and **c** DiR-labeled PEG-Lipo observed by TEM.

## Data Availability

All data generated or analysed during this study are included in this published article and can be shared by contacting the corresponding authors.

## Abbreviations

ACGs: Annonaceous acetogenins
DLC: Drug loading content
DiR: 1,1′-Dioctadecyl-3,3,3′,3′-tetramethylindotricarbocyanine iodide
EE: Encapsulation efficiency
IC50: 50% Inhibitory concentration
LDLC: Low drug loading content
MPS: Mononuclear phagocyte system
PDI: Polydispersity index
PEG-ACGs-Lipo: ACGs-loaded PEGylated liposomes
PEG-Lipo: PEGylated liposomes without active molecule
PTX: Paclitaxel
RTTI: Relative tumor targeting index
SPC: Soybean phospholipids
TMZ: Temozolomide
TAMs: Tumor associated macrophages
TIR: Tumor inhibition rate

## References

[1] Campoccia D, Ravaioli S, Santi S, Mariani V, Santarcangelo C, De Filippis A, Montanaro L, Arciola CR, Daglia M (2021). Exploring the anticancer effects of standardized extracts of poplar-type propolis: in vitro cytotoxicity toward cancer and normal cell lines. Biomed Pharmacother.

[2] Esfandiari Nazzaro E, Sabei FY, Vogel WK, Nazari M, Nicholson KS, Gafken PR, Taratula O, Taratula O, Davare MA, Leid M (2021). Discovery and validation of a compound to target Ewing's sarcoma. Pharmaceutics.

[3] Bent EH, Millan-Barea LR, Zhuang I, Goulet DR, Frose J, Hemann MT (2021). Microenvironmental IL-6 inhibits anti-cancer immune responses generated by cytotoxic chemotherapy. Nat Commun.

[4] Wen Q, Zhang Y, Muluh TA, Xiong K, Wang B, Lu Y, Wu Z, Liu Y, Shi H, Xiao S (2021). Erythrocyte membrane-camouflaged gefitinib/albumin nanoparticles for tumor imaging and targeted therapy against lung cancer. Int J Biol Macromol.

[5] El Nashar EM, Alghamdi MA, Alasmari WA, Hussein MMA, Hamza E, Taha RI, Ahmed MM, Al-Khater KM, Abdelfattah-Hassan A (2021). Autophagy promotes the survival of adipose mesenchymal stem/stromal cells and enhances their therapeutic effects in cisplatin-induced liver injury via modulating TGF-beta1/Smad and PI3K/AKT signaling pathways. Cells.

[6] Oldenburg M, Ruchel N, Janssen S, Borkhardt A, Gossling KL (2021). The microbiome in childhood acute lymphoblastic leukemia. Cancers (Basel).

[7] Li R, Gong X, Hong C, Wang H, Chen Y, Tan K, Liu X, Wang F (2021). An efficient photochemotherapy nanoplatform based on the endogenous biosynthesis of photosensitizer in macrophage-derived extracellular vesicles. Biomaterials.

[8] Zhou K, Chen X, Zhang L, Yang Z, Zhu H, Guo D, Su R, Chen H, Li H, Song P (2021). Targeting peripheral immune organs with self-assembling prodrug nanoparticles ameliorates allogeneic heart transplant rejection. Am J Transplant.

[9] Xu J, Salari A, Wang Y, He X, Kerr L, Darbandi A, de Leon AC, Exner AA, Kolios MC, Yuen D (2021). Microfluidic generation of monodisperse nanobubbles by selective gas dissolution. Small.

[10] Subhan MA, Torchilin VP (2019). Efficient nanocarriers of siRNA therapeutics for cancer treatment. Transl Res.

[11] Wang X, Wu M, Zhang X, Li F, Zeng Y, Lin X, Liu X, Liu J (2021). Hypoxia-responsive nanoreactors based on self-enhanced photodynamic sensitization and triggered ferroptosis for cancer synergistic therapy. J Nanobiotechnol.

[12] Zhao M, Yang X, Fu H, Chen C, Zhang Y, Wu Z, Duan Y, Sun Y (2021). Immune/hypoxic tumor microenvironment regulation-enhanced photodynamic treatment realized by pH-responsive phase transition-targeting nanobubbles. ACS Appl Mater Interfaces.

[13] Hwang J, Zhang W, Park HB, Yadav D, Jeon YH, Jin JO (2021). Escherichia coli adhesin protein-conjugated thermal responsive hybrid nanoparticles for photothermal and immunotherapy against cancer and its metastasis. J Immunother Cancer.

[14] Li Z, Yang G, Han L, Wang R, Gong C, Yuan Y (2021). Sorafenib and triptolide loaded cancer cell-platelet hybrid membrane-camouflaged liquid crystalline lipid nanoparticles for the treatment of hepatocellular carcinoma. J Nanobiotechnol.

[15] Shang Q, Zhou S, Zhou Z, Jiang Y, Luan Y (2021). Dual cancer stem cell manipulation to enhance phototherapy against tumor progression and metastasis. J Control Release.

[16] Bao W, Liu M, Meng J, Liu S, Wang S, Jia R, Wang Y, Ma G, Wei W, Tian Z (2021). MOFs-based nanoagent enables dual mitochondrial damage in synergistic antitumor therapy via oxidative stress and calcium overload. Nat Commun.

[17] Peng T, Huang Y, Feng X, Zhu C, Yin S, Wang X, Bai X, Pan X, Wu C (2021). TPGS/hyaluronic acid dual-functionalized PLGA nanoparticles delivered through dissolving microneedles for markedly improved chemo-photothermal combined therapy of superficial tumor. Acta Pharm Sin B.

[18] Liu P, Gao C, Chen H, Vong CT, Wu X, Tang X, Wang S, Wang Y (2021). Receptor-mediated targeted drug delivery systems for treatment of inflammatory bowel disease: opportunities and emerging strategies. Acta Pharm Sin B.

[19] Chen J, Zhang Y, Zhao L, Zhang Y, Chen L, Ma M, Du X, Meng Z, Li C, Meng Q (2021). Supramolecular drug delivery system from macrocycle-based self-assembled amphiphiles for effective tumor therapy. ACS Appl Mater Interfaces.

[20] Dai J, Wu M, Wang Q, Ding S, Dong X, Xue L, Zhu Q, Zhou J, Xia F, Wang S (2021). Red blood cell membrane-camouflaged nanoparticles loaded with AIEgen and Poly(I:C) for enhanced tumoral photodynamic-immunotherapy. Natl Sci Rev.

[21] Vijayaraghavan S, Lipfert L, Chevalier K, Bushey BS, Henley B, Lenhart R, Sendecki J, Beqiri M, Millar HJ, Packman K (2020). Amivantamab (JNJ-61186372), an Fc enhanced EGFR/cMet bispecific antibody, induces receptor downmodulation and antitumor activity by monocyte/macrophage trogocytosis. Mol Cancer Ther.

[22] Ackerman SE, Pearson CI, Gregorio JD, Gonzalez JC, Kenkel JA, Hartmann FJ, Luo A, Ho PY, LeBlanc H, Blum LK (2021). Immune-stimulating antibody conjugates elicit robust myeloid activation and durable antitumor immunity. Nat Cancer.

[23] Furuuchi K, Rybinski K, Fulmer J, Moriyama T, Drozdowski B, Soto A, Fernando S, Wilson K, Milinichik A, Dula ML (2021). Antibody-drug conjugate MORAb-202 exhibits long-lasting antitumor efficacy in TNBC PDx models. Cancer Sci.

[24] Zhu M, Sheng Z, Jia Y, Hu D, Liu X, Xia X, Liu C, Wang P, Wang X, Zheng H (2017). Indocyanine green-holo-transferrin nanoassemblies for tumor-targeted dual-modal imaging and photothermal therapy of glioma. ACS Appl Mater Interfaces.

[25] Kucharz K, Kristensen K, Johnsen KB, Lund MA, Lonstrup M, Moos T, Andresen TL, Lauritzen MJ (2021). Post-capillary venules are the key locus for transcytosis-mediated brain delivery of therapeutic nanoparticles. Nat Commun.

[26] Wu P, Sun Y, Dong W, Zhou H, Guo S, Zhang L, Wang X, Wan M, Zong Y (2019). Enhanced anti-tumor efficacy of hyaluronic acid modified nanocomposites combined with sonochemotherapy against subcutaneous and metastatic breast tumors. Nanoscale.

[27] Zhang Y, Xia Q, Wu T, He Z, Li Y, Li Z, Hou X, He Y, Ruan S, Wang Z (2021). A novel multi-functionalized multicellular nanodelivery system for non-small cell lung cancer photochemotherapy. J Nanobiotechnol.

[28] Chiesa E, Greco A, Riva F, Dorati R, Conti B, Modena T, Genta I (2021). Hyaluronic acid-based nanoparticles for protein delivery: systematic examination of microfluidic production conditions. Pharmaceutics.

[29] Pirollo KF, Chang EH (2008). Does a targeting ligand influence nanoparticle tumor localization or uptake?. Trends Biotechnol.

[30] Kirpotin DB, Drummond DC, Shao Y, Shalaby MR, Hong K, Nielsen UB, Marks JD, Benz CC, Park JW (2006). Antibody targeting of long-circulating lipidic nanoparticles does not increase tumor localization but does increase internalization in animal models. Cancer Res.

[31] Mikhail AS, Allen C (2009). Block copolymer micelles for delivery of cancer therapy: transport at the whole body, tissue and cellular levels. J Control Release.

[32] Cheng YH, He C, Riviere JE, Monteiro-Riviere NA, Lin Z (2020). Meta-analysis of nanoparticle delivery to tumors using a physiologically based pharmacokinetic modeling and simulation approach. ACS Nano.

[33] Dai Q, Wilhelm S, Ding D, Syed AM, Sindhwani S, Zhang Y, Chen YY, MacMillan P, Chan WCW (2018). Quantifying the ligand-coated nanoparticle delivery to cancer cells in solid tumors. ACS Nano.

[34] Farrag NS, Shetta A, Mamdouh W (2021). Green tea essential oil encapsulated chitosan nanoparticles-based radiopharmaceutical as a new trend for solid tumor theranosis. Int J Biol Macromol.

[35] Ouyang B, Poon W, Zhang YN, Lin ZP, Kingston BR, Tavares AJ, Zhang Y, Chen J, Valic MS, Syed AM (2020). The dose threshold for nanoparticle tumour delivery. Nat Mater.

[36] Li H, Li Y, Ao H, Bi D, Han M, Guo Y, Wang X (2018). Folate-targeting annonaceous acetogenins nanosuspensions: significantly enhanced antitumor efficacy in HeLa tumor-bearing mice. Drug Deliv.

[37] Ao H, Li HW, Lu LK, Fu JX, Han MH, Guo YF, Wang XT (2021). Sensitive tumor cell line for annonaceous acetogenins and high therapeutic efficacy at a low dose for choriocarcinoma therapy. J Biomed Nanotechnol.

[38] Hong J, Li Y, Xiao Y, Li Y, Guo Y, Kuang H, Wang X (2016). Annonaceous acetogenins (ACGs) nanosuspensions based on a self-assembly stabilizer and the significantly improved anti-tumor efficacy. Colloids Surf B Biointerfaces.

[39] Hong J, Li Y, Li Y, Xiao Y, Kuang H, Wang X (2016). Annonaceous acetogenins nanosuspensions stabilized by PCL-PEG block polymer: significantly improved antitumor efficacy. Int J Nanomed.

[40] Hong J, Sun Z, Li Y, Guo Y, Liao Y, Liu M, Wang X (2017). Folate-modified Annonaceous acetogenins nanosuspensions and their improved antitumor efficacy. Int J Nanomed.

[41] Yuan F, Bai G, Chen Y, Miao Y, Chen J, Li X (2015). Structure–activity relationships of diverse ACGs against multidrug resistant human lung cancer cell line A549/Taxol. Bioorg Med Chem Lett.

[42] Lazaro-Ibanez E, Faruqu FN, Saleh AF, Silva AM, Tzu-Wen Wang J, Rak J, Al-Jamal KT, Dekker N (2021). Selection of fluorescent, bioluminescent, and radioactive tracers to accurately reflect extracellular vesicle biodistribution in vivo. ACS Nano.

[43] Wang QL, Zhuang X, Sriwastva MK, Mu J, Teng Y, Deng Z, Zhang L, Sundaram K, Kumar A, Miller D (2018). Blood exosomes regulate the tissue distribution of grapefruit-derived nanovector via CD36 and IGFR1 pathways. Theranostics.

[44] Berninger MT, Mohajerani P, Kimm M, Masius S, Ma X, Wildgruber M, Haller B, Anton M, Imhoff AB, Ntziachristos V (2017). Fluorescence molecular tomography of DiR-labeled mesenchymal stem cell implants for osteochondral defect repair in rabbit knees. Eur Radiol.

[45] Caracciolo G (2015). Liposome-protein corona in a physiological environment: challenges and opportunities for targeted delivery of nanomedicines. Nanomedicine.

[46] Moosavian SA, Sahebkar A (2019). Aptamer-functionalized liposomes for targeted cancer therapy. Cancer Lett.

[47] Ashrafzadeh MS, Akbarzadeh A, Heydarinasab A, Ardjmand M (2020). In vivo glioblastoma therapy using targeted liposomal cisplatin. Int J Nanomed.

[48] Gaillard PJ, Appeldoorn CC, Dorland R, van Kregten J, Manca F, Vugts DJ, Windhorst B, van Dongen GA, de Vries HE, Maussang D (2014). Pharmacokinetics, brain delivery, and efficacy in brain tumor-bearing mice of glutathione pegylated liposomal doxorubicin (2B3–101). PLoS ONE.

[49] Ziemys A, Yokoi K, Kojic M (2015). Capillary collagen as the physical transport barrier in drug delivery to tumor microenvironment. Tissue Barriers..

[50] Sheng Q, Li T, Tang X, Zhao W, Guo R, Cun X, Zang S, Zhang Z, Li M, He Q (2021). Comprehensively enhanced delivery cascade by transformable beaded nanofibrils for pancreatic cancer therapy. Nanoscale.

[51] Du JZ, Li HJ, Wang J (2018). Tumor-acidity-cleavable maleic acid amide (TACMAA): a powerful tool for designing smart nanoparticles to overcome delivery barriers in cancer nanomedicine. Acc Chem Res.

[52] Xu CF, Zhang HB, Sun CY, Liu Y, Shen S, Yang XZ, Zhu YH, Wang J (2016). Tumor acidity-sensitive linkage-bridged block copolymer for therapeutic siRNA delivery. Biomaterials.

