# Supplementary material for: Enhanced tumor accumulation and therapeutic efficacy of liposomal drugs through over-threshold dosing
Source: J Nanobiotechnology. 2022 Mar 15;20:137. doi: 10.1186/s12951-022-01349-1 (PMC8922779; doi:10.1186/s12951-022-01349-1)
Supplement: Supplementary file 1 — Additional file 1: Fig. S1. The chemical structures of a squamocin and b bullatacin (the two major components in ACGs). Fig. S2. Characterization of two DiR-labeled liposomes. a The particle size distribution of two DiR-labeled liposomes. The morphology of b DiR-labeled PEG-ACGs-Lipo and c DiR-labeled PEG-Lipo observed by TEM. [file 12951_2022_1349_MOESM1_ESM.docx]

Supplementary material for

**Enhanced tumor accumulation and therapeutic efficacy of liposomal drugs through** **over-threshold dosing**

Hui Ao^1^, Zhuo Wang^2^, Likang Lu^1^, Hongwei Ma^2^, Haowen Li^1^, Jingxin Fu^1^, Manzhen Li^1^, Meihua Han^1^, Yifei Guo^1^ and Xiangtao Wang^1^*

^1^Institute of Medicinal Plant Development, Chinese Academy of Medical Sciences & Peking Union Medical College, No. 151, Malianwa North Road, Haidian District, Beijing, 100193, PR China.

^2^ College of Pharmacy, Heilongjiang University of Chinese Medicine, No. 24, Heping Road, Xiangfang District, Harbin, 150040, PR China.

*Corresponding author: Xiangtao Wang, Email: [xtaowang@163.com](mailto:xtaowang@163.com)

**The Supplementary material includes:**

**Fig. S1.** The chemical structures of **a** squamocin and **b** bullatacin (the two major components in ACGs).

**Fig. S2.** Characterization of two DiR-labeled liposomes. **a** The particle size distribution of two DiR-labeled liposomes. The morphology of **b** DiR-labeled PEG-ACGs-Lipo and **c** DiR-labeled PEG-Lipo observed by TEM.

**Supplemental Figures**


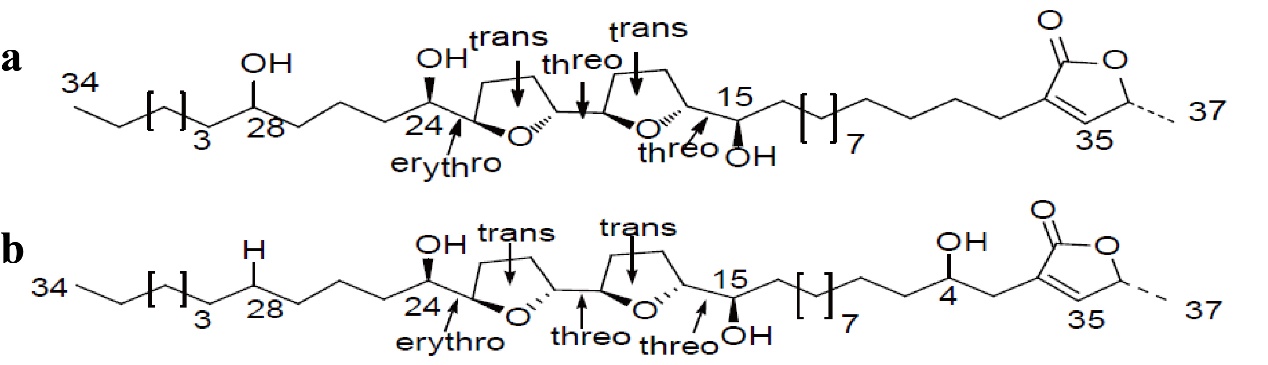


**Fig. S1** The chemical structures of **a** squamocin and **b** bullatacin (the two major components in ACGs).


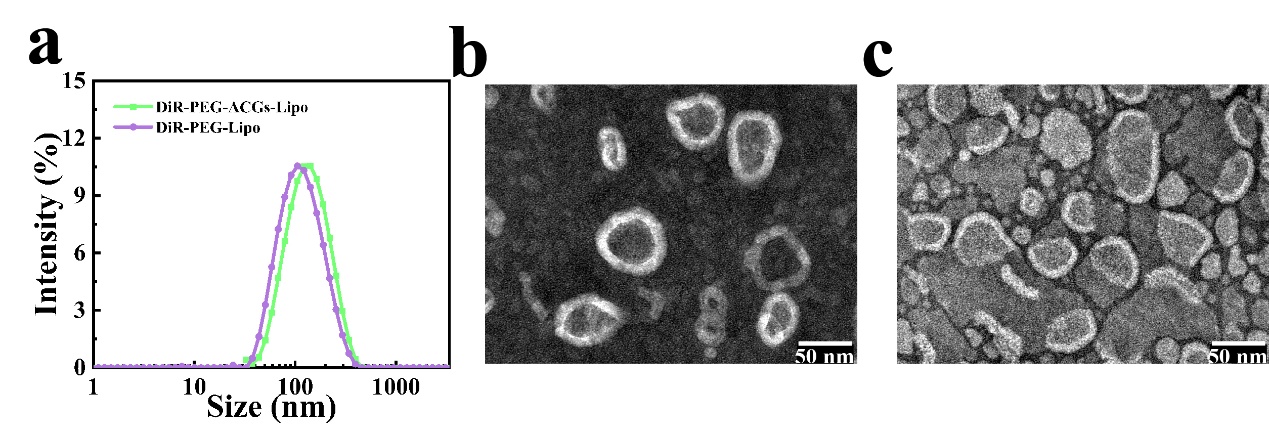


**Fig. S2** Characterization of two DiR-labeled liposomes. **a** The particle size distribution of two DiR-labeled liposomes. The morphology of **b** DiR-labeled PEG-ACGs-Lipo and **c** DiR-labeled PEG-Lipo observed by TEM.
